# Giant J (Osborn) Wave due to Bonsai Abuse: Comments on Clinical Practice

**DOI:** 10.4274/balkanmedj.2015.1233

**Published:** 2017-01-05

**Authors:** Murat Yalçın, Mustafa Aparcı, Murat Eroğlu, Zafer Işılak, Namık Özmen

**Affiliations:** 1 Department of Cardiology, Sultan Abdülhamid Han Training and Research Hospital, İstanbul, Turkey; 2 Department of Cardiology, Kasımpaşa Military Hospital, İstanbul, Turkey; 3 Department of Emergency Medicine, Sultan Abdülhamid Han Training and Research Hospital, İstanbul, Turkey

**Keywords:** Osborn, cannabis, arrhythmia

## Abstract

**Background:**

Osborn wave, typically associated with hypothermia, is currently referred to as one of the J wave syndromes due to its clinical potential to develop lethal cardiac arrhythmia; it may rarely be observed in a non-hypothermic setting such as cannabis abuse.

**Case Report:**

In this paper, we presented two young cases who presented to the emergency services with unconsciousness, drowsiness, and hypoxia, and also J wave on electrocardiography (ECG) due to Bonsai abuse.

**Conclusion:**

Osborn wave may be a significant criterion to initiate close monitoring in a coronary care unit, with supportive treatment and mechanical ventilation as necessary in those patients who abuse Bonsai.

Cannabis and Bonsai abuse has been increasing among adolescents and teenagers worldwide (1). Bonsai is a synthetically-derived illicit drug which mimics the effects of cannabis and is known as “spice” in European countries. The increased prevalence of Bonsai and cannabis abuse results in increased fatalities due to cardiovascular events in the young population (2). Myocardial ischemia or infarction even with normal coronary arteries is one of the frequent findings associated with cardiovascular death due to cannabis and bonsai (3,4).

Osborn wave, which was first described in 1953 and is supposed to be a physiologic effect of hypothermia, is currently referred to as one of the J wave syndromes due to its clinical potential to develop lethal cardiac arrhythmia (5). Numerous clinical entities and various patterns of electrocardiographic J wave changes have been reported to be associated with varying levels of risk for the development of malignant arrhythmia (5).

We presented two young cases who admitted to emergency services with unconsciousness, drowsiness, and hypoxia, and also J wave on electrocardiography (ECG) due to Bonsai abuse.

## CASE PRESENTATIONS

### CASE 1

**Figure 1:**
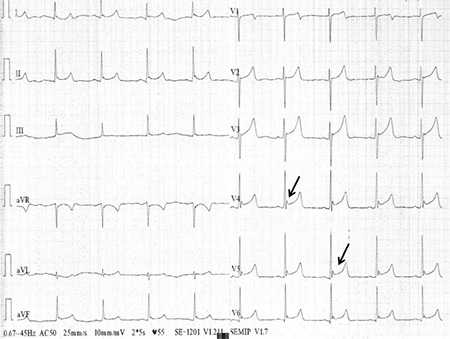
Electrocardiography recorded on admission; Osborn waves can be seen in leads II, III, and aVF, and prominently on V3-V6 derivations (black arrow).

**Figure 2:**
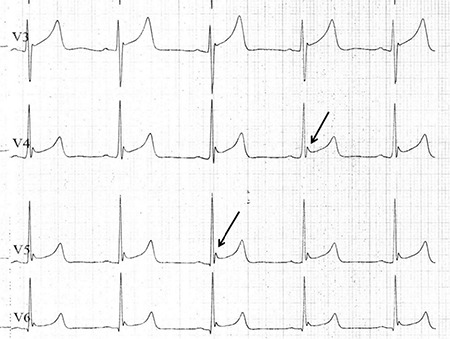
Electrocardiography recorded on admission; Zoomed-in view of the V3-V6 derivations (black arrow).

**Figure 3. a, b:**
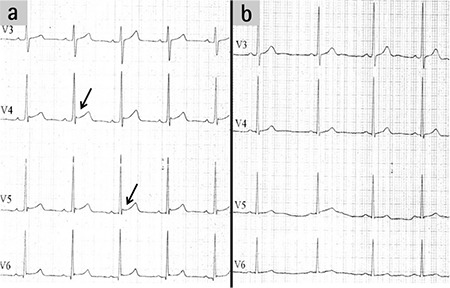
Electrocardiography recorded after an 8 hour period: Osborn waves were still present (black arrow) (a). ECG recorded after a 36 hour period: Osborn waves had disappeared (b).

A 21 year-old male was transferred to the emergency room with confusion and loss of consciousness following several hours of bonsai abuse. In his physical examination, his blood pressure was 110/70 mmHg, heart rate was 68 beats per minute, and body temperature was 34.4 °C. Breathing was shallow, at a rate of 8 breaths per minute. Pulse oximetry revealed the oxygen saturation to be 81%. Arterial blood gas analysis was interpreted as acute respiratory acidosis with reduced pH to 7.23 and elevated PaCO_2_ up to 67 mmHg. Serum levels of biochemistry parameters including blood urea nitrogen, creatinine and electrolytes, all of which were within normal ranges. On the 12 lead ECG (Cardiofax; Nihon Kohden, Japan), a deflection of J wave-Osborn wave was globally observed on leads I, aVL, II, III, and aVF, and prominently on V_2_-V_6_ derivations, which may be described as a Type 3 pattern; early repolarization on all derivations with the highest risk for malignant arrhythmia (Figure 1, 2). The patient was intubated and ventilated mechanically and then transferred to the intensive care unit. Although the patient had been warmed up to 37.1 °C over an 8 hour period, it was observed that the Osborn wave did not recover on ECG (Figure 3a). After a 36 hour period, the patient was weaned from mechanical ventilation (Drager EviteXL; Drägerwerk, Germany) and extubated following an improvement of blood gas analysis and unconsciousness. We observed that the Osborn wave had disappeared on ECG at the end of the 36 hour period (Figure 3b). The patient was consulted by the psychiatry clinic and discharged after an additional 3 days follow-up by the cardiology service. A written informed consent was obtained after the patient recovered.

### CASE 2

**Figure 4:**
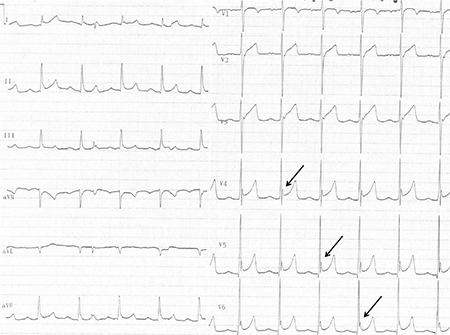
Electrocardiography recorded on admission; Osborn waves can be seen in leads II, III, and aVF, and prominently on V3-V6 derivations (black arrow).

**Figure 5:**
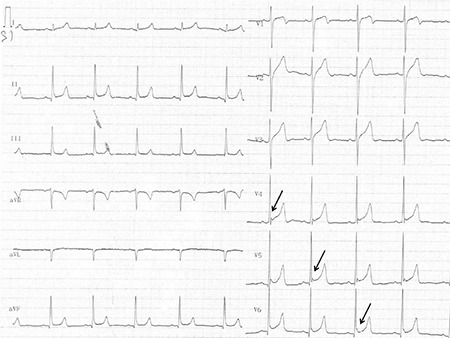
Electrocardiography recorded after a 12 hour period: Osborn waves were still present (black arrow).

A 20 year-old male was transferred to the emergency room with confusion after bonsai abuse. On physical examination, his blood pressure was 120/75 mmHg, heart rate was 75 beats per minute, and body temperature was 36.5 °C. The breathing rate was 10 breaths per minute and the oxygen saturation was 90% on pulse oximeter. Arterial blood gas analysis and also serum biochemistry panel were within normal ranges. The J wave-Osborn wave was similar on leads II, III, and aVF, and prominently on V3-V6 derivations, and was described as a Type 2 pattern (Figure 4). The patient was followed-up for one-day in the coronary care unit and supported by nasal oxygen and intravenous serum saline administration. J wave elevation persisted for at least 12 hours and then disappeared after a 24 hour period (Figure 5). Following an uncomplicated observation period of 24 hours, the patient was transferred to the cardiology service and monitored for three days. Thereafter, he was discharged after consulting the psychiatry clinic. A written informed consent was obtained after the patient recovered.

## DISCUSSION

Although the "Osborn Wave" was initially supposed to be pathognomonic for hypothermia, it was reported to be associated with numerous critical clinical conditions such as hypercapnia, brain injury, and subarachnoid hemorrhage, cardiopulmonary arrest during therapeutic hypothermia, and various electrolyte abnormalities, as well as vasospastic angina (6,7,8,9). It is currently accepted as the ECG finding of J Wave syndromes such as Brugada syndrome and early repolarization syndromes which may potentially result in idiopathic ventricular fibrillation (5).

Those cases may be the first to present with unconsciousness, respiratory acidosis and coexisting Osborn wave due to Bonsai abuse. Their poor clinical condition and respiratory failure might have worsened unless supportive parenteral and respiratory intervention had been initiated. Development of Osborn wave upon the centrally induced respiratory acidosis and its disappearance spontaneously following supportive parenteral and respiratory interventions may be clinically significant indicators prompting the administration of supportive interventions in patients who abused Bonsai, cannabis, etc. and presented with critical illness and unconsciousness. Osborn wave-elevation of ST segment at J point may be the initial stage that precedes sudden cardiac death due to malignant ventricular arrhythmia in young bonsai abusers who have an increased prevalence of sudden cardiac death. Osborn wave may be a clinical indicator for close hemodynamic monitoring and supportive treatment and continuation of supportive therapy until the ECG changes return to normal.

It may be suggested that Bonsai and similar synthetically-derived illicit drugs may cause sudden cardiac death syndromes through drug induced sodium ion current abnormalities leading to malignant ventricular arrhythmia as well as drug induced QT prolongation and sudden cardiac deaths. Ambulatory rhythm monitoring in patients who abuse Bonsai and other substances may allow the detection of paroxysmal drug-induced ECG changes and also sustained/non-sustained ventricular arrhythmia. Although no anti-arrhythmic drug had been prophylactically given to our patients, findings on ambulatory rhythm monitoring in further cases may revive the option of prophylactic anti-arrhythmic drug use in those patients and earlier defibrillation or cardioversion using either automated or manual external defibrillators in the management of young cases who had abused Bonsai and presented with unconsciousness. Although it may be suggested that the hypothermia in the first case may be a confounding factor for Osborn wave, its development was reported to be a strong predictor for recurrent ventricular fibrillations, even during therapeutic hypothermia (9). Central inhibition of ventilatory drive resulting in respiratory acidosis may be one of the complications of Bonsai and its derivatives. Acute respiratory acidosis represented by hypercapnia and reduced pH might have developed due to Bonsai-induced central respiratory depression. Ventilatory support including mechanical ventilation or noninvasive positive pressure ventilation until the acidosis, hypercapnia, and hypoxia improve and also Osborn wave disappear may be the lifesaving therapeutic measures in the management of complicated young Bonsai abusers.

In conclusion, the prevalence of young subjects who abuse Bonsai or synthetically derived illicit drugs is increasing. Therefore, sudden cardiac deaths or admittance to emergency rooms in a comatose state is inevitably increasing. Osborn wave may be a significant criterion to initiate close monitoring in a coronary care unit, supportive treatment, and mechanical ventilation as necessary in those patients who abuse Bonsai and become comatose. Osborn wave may be an ECG sign preceding an electrical storm in those patients unless they are managed earlier and treated effectively.
